# Moderators of the Effects of a Digital Parenting Intervention on Child Conduct and Emotional Problems Implemented During the COVID-19 Pandemic: Results From a Secondary Analysis of Data From the Supporting Parents and Kids Through Lockdown Experiences (SPARKLE) Randomized Controlled Trial

**DOI:** 10.2196/53864

**Published:** 2024-10-08

**Authors:** Nikola Pokorna, Melanie Palmer, Oliver Pearson, Nicholas Beckley-Hoelscher, James Shearer, Katarzyna Kostyrka-Allchorne, Olly Robertson, Marta Koch, Petr Slovak, Crispin Day, Sarah Byford, Polly Waite, Cathy Creswell, Edmund J S Sonuga-Barke, Kimberley Goldsmith

**Affiliations:** 1 Institute of Psychiatry, Psychology & Neuroscience King’s College London London United Kingdom; 2 Department of Psychology, School of Biological and Behavioural Sciences, Queen Mary University of London London United Kingdom; 3 Department of Experimental Psychology University of Oxford Oxford United Kingdom

**Keywords:** parenting, intervention, smartphone app, randomized controlled trial, COVID-19 pandemic, moderators, conduct problems, emotional problems

## Abstract

**Background:**

A smartphone app, Parent Positive, was developed to help parents manage their children’s conduct and emotional problems during the COVID-19 pandemic. A randomized controlled trial, Supporting Parents and Kids Through Lockdown Experiences (SPARKLE), found Parent Positive to be effective in reducing children’s emotional problems. However, app effectiveness may be influenced by a range of child, family, socioeconomic, and pandemic-related factors.

**Objective:**

This study examined whether baseline factors related to the child, family, and socioeconomic status, as well as pandemic-related disruption circumstances, moderated Parent Positive’s effects on child conduct and emotional problems at 1- and 2-month follow-up.

**Methods:**

This study was a secondary exploratory analysis of SPARKLE data. The data set included 646 children (4-10 years of age) with parents randomized to either Parent Positive (n=320) or follow-up as usual (n=326). Candidate baseline moderators included child age, gender, attention-deficit/hyperactivity disorder symptoms, parental psychological distress, family conflict, household income, employment status, household overcrowding, and pandemic-related disruption risk (ie, homeschooling, lockdown status, and isolation status). Child conduct and emotional problem outcomes measured at 1- (T2) and 2-months (T3) post randomization were analyzed using linear mixed-effects analysis of covariance models adjusting for baseline (T1) measure of outcome and including intervention and intervention by time point interaction terms allowing for different effects at the 2 time points. Moderation of intervention effects by baseline factors was assessed by replacing the intervention by time interaction terms with intervention by time point by baseline moderator interaction terms.

**Results:**

Child gender was a significant moderator of the Parent Positive versus follow-up as usual effect on emotional problems (*B*=0.72, 95% CI 0.12-1.33; *P*=.02). Specifically, the effect of Parent Positive was close to significant (T2: *B*=–0.41, 95% CI –0.82 to 0.0004; *P*=.05) or significant (T3: *B*=–0.76, 95% CI –1.22 to –0.30; *P*<.001) in males only when compared with females, and males experienced a significantly larger reduction in emotional problems than females in the Parent Positive arm at the 2-month post randomization time point. None of the other investigated baseline factors moderated effects on emotional problems, and no factors moderated effects on conduct problems.

**Conclusions:**

This study highlights Parent Positive’s potential for effectively reducing emotional problems in primary school-aged male children across a wide range of families. However, due to limited variability in the demographic background of the families, cautious interpretation is required, and replications are necessary in diverse samples with longer follow-up times.

**Trial Registration:**

ClinicalTrials.gov NCT04786080; https://clinicaltrials.gov/ct2/show/NCT04786080

## Introduction

### Background

Child conduct and emotional problems are a public health concern due to their negative impact on individuals, their families, and society [1]. Conduct problems are characterized by oppositional, disruptive, or aggressive behaviors [2], while emotional problems include anxiety and depression [3]. Conduct and emotional problems frequently co-occur [4] and can disrupt daily functioning, mental health, and social interactions [5]. If left untreated, these issues may elevate the likelihood of mental disorders, substance use, and delinquency in childhood and adolescence [6], as well as criminality in adulthood [7]. Children were particularly vulnerable to mental health difficulties during the global COVID-19 pandemic, likely due to COVID-19 mitigation strategies imposed on families by governments (eg, lockdowns, social isolation, and school closures) [8]. In a large cohort study conducted in the United Kingdom—COVID-19: Supporting Parents, Adolescents, and Children During Epidemics (Co-SPACE) [9]—lockdowns coincided with increased conduct and emotional problems in participating primary school-aged children.

Child conduct and emotional problems can be effectively reduced by behavioral parenting training that aims to enhance parenting practices [10]. However, financial costs, lack of access to transport, and stigma limit participation in these interventions, which are typically delivered face-to-face [11], and there were particular access issues during lockdowns. To increase accessibility, such interventions have shifted to smartphone app–based delivery formats [12,13]. Recently, Kostyrka-Allchorne et al [14] developed an evidence-based smartphone app, Parent Positive, that aimed to support parents and carers (henceforth referred to as parents) within the general population during the COVID-19 pandemic. Results from a randomized controlled trial (RCT)—Supporting Parents and Kids Through Lockdown Experiences (SPARKLE) evaluating the effects of Parent Positive versus a follow-up as usual arm (FAU)—showed that Parent Positive significantly reduced child emotional, but not conduct, problems [15] at 1- and 2-month follow-ups as compared with FAU.

While there were benefits of Parent Positive for the whole sample, as common for many interventions, there may be considerable variation in its benefits between individuals. In this case, it becomes important to identify factors influencing intervention outcomes to support a more targeted intervention approach. The literature describes such factors as intervention moderators, as they interact with the intervention and improve outcomes for some over others [16,17]. Exploring these moderators could improve understanding of which interventions are beneficial for whom, enabling clinicians to tailor recommendations to the most effective interventions [17,18]. We note that 1 moderation hypothesis was evaluated as part of the main SPARKLE trial analysis and was published in the main results paper: the effect of Parent Positive on conduct problems did not differ by levels of conduct problems at baseline [15].

The examination of moderator effects on child outcomes in previous studies of parenting interventions has been somewhat limited. Rather than conducting moderation analyses, some studies have primarily focused on assessing associations between various factors and outcomes only within groups exposed to interventions, or across the trial sample as a whole [16,19]. It is essential to distinguish this approach from moderation analyses, which involve investigating whether the effect of an intervention differs by the level of baseline characteristics or factors [16]. In this regard, there has been a somewhat limited number of studies explicitly exploring moderation effects in parenting interventions.

Most of the research on moderating factors in parenting interventions has examined how child, family, and socioeconomic factors influence the effectiveness of these interventions in addressing child conduct problems. Overall, the evidence regarding which moderators of parenting interventions are impactful on child conduct problems is somewhat mixed. The consensus from most meta-analyses suggests that child age [20-22] does not significantly moderate the effects of parenting interventions. Regarding child gender, while a meta-analysis found that boys benefitted more than girls from a parenting intervention [20], 2 RCT studies did not find any moderation effects related to child gender [23,24]. In the context of attention-deficit/hyperactivity disorder (ADHD) symptoms, multiple meta-analyses did not identify ADHD as a significant moderator of parenting intervention effects on child conduct problems [20,25,26].

Recent systematic reviews [19,27] exploring moderating effects of family risk factors, such as parental psychopathology, life stress, and family conflicts, reported no impact of these factors on effectiveness of parenting interventions for child conduct problems. Conversely, 1 meta-analysis indicated that children exhibited greater improvements when their mothers displayed elevated levels of depressive symptoms [26].

When examining socioeconomic status as a moderator in parenting intervention studies, the results have been less consistent. Most research indicates that these interventions maintain their effectiveness across different socioeconomic backgrounds [19,27], with recent meta-analyses and reviews [20,25] finding no significant moderation effects of socioeconomic characteristics on child conduct problems. However, other studies have suggested that socioeconomic disadvantage may lead to less positive child outcomes [21,28].

However, several of these moderator analyses faced limitations attributed to small sample sizes (ie, fewer than 70 participants in each arm of an RCT study [23]), likely lacking power to detect an effect that exists (type II error) [19]. In addition, there were variations in intervention components, including content, delivery format, length, and therapist contact [19]. These variations may have contributed to a lack of clear results, as what moderates intervention effects on outcomes likely varies across different interventions [19]. Furthermore, while previous research on parenting interventions has primarily examined moderation effects of child, family, and socioeconomic characteristics [19], recent pandemic-related disruptions could also influence intervention outcomes on children during the pandemic due to associated challenges. Consequently, the negative impact of mitigation policies [29] could influence how children and families respond to parenting interventions. For example, joint confinement and isolation from support networks, with parents often having to manage competing homeschooling and work demands have placed great pressure on parent-child relationships. In turn, it could have been more difficult for parents to engage within a self-directed universal intervention. This highlights the importance of examining moderating factors of a digital intervention within the context of the COVID-19 pandemic, even if previously studied and not found to be significant moderators, as the distinctive circumstances may yield diverse outcomes.

### Objectives

This study aimed to test whether child, family, socioeconomic, and pandemic-related circumstances at baseline moderated Parent Positive effects on child conduct and emotional problems at 1- and 2-month follow-up in the SPARKLE trial. Given the inconsistent previous findings regarding moderators of parenting interventions and the novelty of the intervention [19], we chose an exploratory approach. The following factors, thought to be potential moderators, were used to construct a multivariate model: child age, gender, ADHD symptoms, parental psychological distress, family conflict, household income, employment status, household overcrowding, and pandemic-related disruption risk.

## Methods

### Study Design

This study was a secondary analysis using repeated-measures data from SPARKLE—a parallel, 2-arm, superiority RCT [14]. SPARKLE tested the effects of Parent Positive compared with FAU. Recruitment commenced in May 2021 during the COVID-19 pandemic and was a Trial within a Cohort [30] embedded in the general population Co-SPACE study [29]. The primary outcome variable was parent-reported levels of child conduct at 1- and 2-month post randomization, with emotional problems measured at the same time points as an important secondary outcome. Throughout the paper, we will refer to the “main analysis” or “main analysis models.” By this, we mean the analysis we used to obtain the main trial results [15]. The main trial outcomes indicated that Parent Positive significantly reduced emotional problems but not conduct problems [15] when compared with FAU at both the 1- and 2-month follow-ups.

### Ethical Considerations

Ethics approval was granted by King’s College London (reference: HR–20/21-21451) and the University of Oxford (SPARKLE reference: R73153/RE001; Co-SPACE reference: R69060/RE001).

Written informed consent was originally provided by participants for the primary trial, covering the use of their data in secondary analyses without requiring additional consent. Participants were fully informed of their rights, including the ability to withdraw from the study at any time without consequence. Participant data were deidentified using unique study IDs, with raw data from the SPARKLE trial deposited in the UK Data Service repository [31]. A data privacy notice was made available on the study website. As compensation for their time, participants received 2 £5 (£1=US $1.26 at time of conversion) web-based shopping vouchers upon completing each follow-up questionnaire. Further information can be found in the SPARKLE protocol [14] and the main trial results article [15].

### Participants

A total of 100% (646/646) children with parents were recruited into SPARKLE between May 19, 2021, and July 26, 2021. Parents were either part of the Co-SPACE cohort before the trial or were eligible to join Co-SPACE (aged 18 years and older, UK residence, informed consent) and SPARKLE after the start of the trial. In addition to the Co-SPACE criteria, SPARKLE participants were required to have a child aged 4-10 years and a smartphone to access Parent Positive (Android: operating system OS 8-9 or higher; Apple: iOS 12-13 or higher).

### Procedures

#### Data Collection

Participants were invited to SPARKLE via a Qualtrics Co-SPACE survey between May 19, 2021, and July 26, 2021, and confirmed their eligibility before giving written e-consent. After completing baseline measures, participants were randomized to either Parent Positive or FAU and automatically informed about their allocated group through the Qualtrics Randomizer function. Participants (320/646, 49.5%) allocated to the Parent Positive arm received free access to Parent Positive from randomization until November 30, 2021. Data were collected at baseline (T1), 1-month (T2), and 2-months (T3) post randomization (via Qualtrics as part of Co-SPACE). Further details about the procedures, including the trial CONSORT (Consolidated Standards of Reporting Trials) diagram, can be found in the main trial results paper [15].

#### Interventions

##### Parent Positive

Parent Positive is an evidence-based self-directed smartphone app delivering support to parents with content organized in 3 zones. The first zone, Parenting Boosters, provided parenting advice on common challenges through 8 animations narrated by celebrity parents. The second zone, Parenting Exchange, provided access to a moderated support platform to connect with other parents and submit questions to experts in the field of child behavior and mental health. The third zone, Parenting Resources, provided parents with links to evidence-based parenting resources. No specific order or time was necessary for accessing the zones. Further details about the intervention can be found in the main trial results paper [15].

##### Follow-Up as Usual

Participants (326/646, 50.5%) randomized to FAU received no intervention during data collection. After their follow-up at T3, they received free access to Parent Positive until November 30, 2021.

### Measures

#### Clinical Outcomes

This section describes the clinical outcomes assessed in the study, focusing on child conduct and emotional problems.

##### Child Conduct and Emotional Problems

The clinical outcomes were parent-reported levels of child conduct and emotional problems measured by the conduct and emotional subscales of the Strengths and Difficulties Questionnaire (SDQ) [32]. Each subscale consists of 5 items with response options ranging from 0 (Not true) to 2 (Certainly true). An overall subscale score was derived from summing the scores of individual items for each subscale, with higher scores indicating greater severity. The internal consistency of the SDQ is good (Cronbach α=0.73) [33].

#### Candidate Moderators

The key candidate baseline moderator variables were child age, gender, ADHD symptoms, parental psychological distress, family conflict, household income, employment status, household overcrowding, and pandemic-related disruption index. The following section provides a detailed description of these child, family, and socioeconomic characteristics.

##### Child Characteristics

###### Child Age and Gender

Child age and gender were provided by parents at baseline in a form asking about background demographics. When responding regarding the gender of their child, 3 parents chose the option “prefer not to say.” Given the limited number of participants in this category, mean imputation was conducted to include these participants. However, when we explored gender-specific moderation effects, data of only the more prevalent categories of males and females were used due to the limited number of participants in the “prefer not to say” category.

###### Child ADHD Symptoms

Child ADHD symptoms were measured by the 5-item hyperactivity-inattention subscale of the SDQ as described in the “Clinical Outcomes” section [32], with response options ranging from 0 (Not true) to 2 (Certainly true). An overall subscale score was derived from summing the scores of individual items, with higher scores indicating greater severity.

##### Family Characteristics

###### Parental Psychological Distress

Parental psychological distress was assessed using the 21-item Depression, Anxiety, and Stress Scale (DASS-21) [34]. The DASS-21 is a self-reported measure of depression, stress, and anxiety symptoms [35], consisting of 3 subscales (7 items each) and response options ranging from 0 (Never) to 3 (Almost always). The total score was multiplied by 2 to obtain a DASS-42 equivalent score [14], ranging from 0 to 126. Higher scores indicate higher severity. It has excellent internal consistency (Cronbach α=0.93) in community samples [36].

###### Family Conflict

The Family Conflict Scale was developed for the Co-SPACE study [29] to assess family conflict. It is a self-reported 3-item measure of arguments and disagreements between family members (parents, parents-children, and siblings). In the Co-SPACE study, family conflict was associated with mental health symptom trajectories during the pandemic [37]. Response options range from 0 (Not at all) to 3 (Completely) and the total score ranges from 0 to 9, with higher scores indicating greater family conflict. The internal consistency in the Co-SPACE sample was Cronbach α>0.54 [14], indicating moderate reliability.

##### Socioeconomic Characteristics

###### Household Income

Household income, collected at baseline, was recoded into 2 categories: less than £29,999 (US $38,600.66) per year and greater than £30,000 (US $38,603.78) per year. To create similar-sized groups, this cutoff was chosen due to less representation of families from lower-income backgrounds.

###### Employment Status

Due to limited numbers, participants’ employment status recorded at baseline was recoded into two categories: (1) not in paid employment, which encompassed parents who were at university, unable to work due to disability, homemakers or full-time parents, unemployed and actively seeking work, or retired, and (2) in paid employment—which included those who were self-employed, working part-time, or engaged in full-time employment.

###### Household Overcrowding

Three demographic variables, collected at baseline, were used to compute the household overcrowding index. The number of adults and children (excluding the parent and child in SPARKLE) was each assessed using a single-item measure, with response options ranging from 1 (None, I am the only adult in the household; None, the child I am answering about is the only child in the household) to 7 (6 or more). To calculate the household overcrowding index, the total number of people was divided by the number of rooms where the family resided. A higher overcrowding index reflects a greater degree of household overcrowding.

##### Pandemic-Related Disruption Characteristics

###### Pandemic-Related Disruption Index

Several variables pertaining to pandemic-related disruption circumstances at baseline were available for this analysis. Among them, 3 variables were selected due to their significant variation in responses and aggregated to calculate a pandemic-related disruption index. The selected variables were lockdown status (no=0; yes=1), isolation status (living life as normal=0; social distancing=1; self-isolating=1), and physical school attendance (yes=0; no=1). Scores ranged from 0 (low disruption) to 3 (high disruption), with higher scores indicating a greater disruption. For the purposes of analysis, the 2 highest scores (2 and 3) were collapsed into 1 category due to the small numbers in these 2 groups. While interpreting values of the pandemic-related disruption index, it is important to consider that the trial commenced during a period when COVID-19–associated lockdown measures had somewhat subsided. Thus, the pandemic-related disruption index exhibited lower values, indicative of a reduced degree of disruption compared with the initial phases of the pandemic.

### Statistical Analysis

The analysis plan was registered prior to completion of the analysis [38]. Data analysis was done using Stata (version 17.0; StataCorp) [39].

#### Base Model

Linear mixed-effects models (LMM) similar to those applied in the main SPARKLE trial analysis were fitted to identify moderators at T2 and T3. The dependent variables were repeated 1-month and 2-month outcome measures (ie, child conduct and emotional problems in separate models), with a random intercept at the participant level to account for the repeated measures. Regardless of observed significance, all models included the main RCT analysis clinical outcome model covariates (the intervention arm, time, the intervention arm by time interaction allowing for different effects at the 2 outcome time points, and baseline outcome measure) and prespecified covariates (child age and gender), as fixed effects [15]. Baseline covariates that predicted missing outcome data, including household income and the overcrowding index for both clinical outcomes, were also incorporated in the analysis, as fixed effects, to be consistent with the main analysis and to make the missing at random assumption of the maximum likelihood estimation algorithm more plausible.

There were several differences between the main trial analysis and the current analysis. In contrast to the main trial analysis, which used household income and the number of adults in the household as separate covariates [15], we used only the overcrowding index. This was because the overcrowding index—which incorporates the number of adults as part of its calculation—was of interest in this study, so we used it rather than number of adults to avoid collinearity. Employment status at baseline predicted missing outcome data of emotional problems and was also incorporated as a fixed-effect covariate in models for emotional problems. In the main analysis, mean imputation [40] was applied to address missing baseline data (including “prefer not to say” responses) for child gender, family conflict, overcrowding index, and physical school attendance (used in the construction of the pandemic-related disruption index). In addition, categorical income was imputed using the mode.

Textbox 1 presents a list of steps and regression equations for the models used in the analysis of child conduct problems. A corresponding list for the analysis of child emotional problems, which includes employment status as a covariate, may be found in the Multimedia Appendix 1.

**A list of steps and regression equations in the analysis of child conduct problems.**

1. Base model (linear mixed-effects models [LMM]):
  - The base model can be represented by the following equation:
    *Y* = β0 + β1 Intervention arm + β2 Time + β3 (Intervention arm × Time) + β4 Baseline child conduct problems + β5 Child age + β6 Child gender + β7 Household income + β8 Overcrowding index + ε
2. Univariate LMM:
  - Fit LMM for each baseline moderator variable of interest as an independent variable in the base model background. Note that we do not know whether a variable is a moderator unless the interaction term in the second equation is significant, but we use that term for consistency. The regression equation example is as follows:
    *Y* = β0 + β1 Intervention arm + β2 Time + β3 (Intervention arm × Time) + β4 Baseline child conduct problems + β5 Child age + β6 Child gender + β7 Household income + β8 Overcrowding index + β9 Moderator + ε
  - Fit LMM models for each baseline variable of interest as an independent moderator to the model background. The regression equation example is as follows:
    *Y* = β0 + β1 Intervention arm + β2 Time + β3 (Intervention arm × Time) + β4 Baseline child conduct problems + β5 Child age + β6 Child gender + β7 Household income + β8 Overcrowding index + β9 Moderator + β10 (Intervention arm × Time × Moderator) + ε
3. Multivariate LMM:
  - Fit forward stepwise LMM with identified main effects from univariate models.
  - Add main effects and 3-way interaction terms in a stepwise manner. The regression equation example is as follows:
    *Y* = β0 + β1 Intervention arm + β2 Time + β3 (Intervention arm × Time) + β4 Baseline child conduct problems + β5 Child age + β6 Child gender + β7 Household income + β8 Overcrowding index + β9 Moderator 𝑥*a* + β10 (Intervention arm × Time × Moderator 𝑥*a*) + β11 Moderator 𝑥*b* + β12 (Intervention arm × Time × Moderator 𝑥*b*) + … + ε

#### Univariate Predictor Mixed-Effects Models

Initially, LMMs were fitted. Each baseline variable of interest, as listed in the objectives section, was included as an independent predictor in the base model described in the previous section. This step allowed for the assessment of the strength of association of each candidate factor individually, determined by likelihood ratio tests and the significance (*P* value) of each variable in turn. Given that the moderating effect of background and pandemic-related factors on intervention effects was the primary focus of this paper [15], the univariate models (ie, adding 1 potential moderator as an independent variable within the base model background) were fitted with intervention arm by time by potential moderator variable interaction terms. Any variable with either a main effect or a main effect and interaction term test *P* value of <.2 in the univariate model was taken forward into consideration in the multivariate models described in the following section. In the analysis of a 3-level categorical variable, pandemic-related disruption index, omnibus tests of significance for the interaction terms were conducted. This methodology involved using a specific test called “testparm” within the Stata software which executed a Wald test.

#### Multivariate Predictor Mixed-Effects Models

Following the univariate analyses, forward stepwise LMMs were fitted to build multivariate independent variable models (ie, might include more than 1 potential moderator as independent variables) in terms of the candidate moderators. Each of the identified main effects (*P*<.2) from the univariate model was manually added one at a time in a descending order with the strongest effect first, as assessed by the largest likelihood ratio test statistic. This enabled us to examine the influence of a range of possible factors simultaneously and assess the contribution of each factor. For each main effect added to the multivariate model, either due to indicating potential 3-way moderation in the univariate model (*P*<.2) or being included as a prespecified covariate, the corresponding 3-way interaction term was added to the model. If no 3-way interaction term was identified in the univariate model (*P*<.2), the factor was included only on its own as a main effect. Main effects and 3-way interaction terms were added in a stepwise manner until no further main effects and interactions terms were found to be statistically significant (*P*<.05). Robust standard errors were used in all final models to account for slight heteroscedasticity of residuals.

## Results

### Sample and Clinical Characteristics

The identical sample was used in this secondary analysis as in the main paper [15]—consisting of 646 parents with eligible children (mean age 7.45, SD 1.67 years; 51.1%, 330/646 male). Given that most variables used were previously described in the main paper, comprehensive information on sample characteristics at baseline is shown in Table S1 in Multimedia Appendix 1 and the 2 clinical outcomes (child conduct and emotional problems) at all time points are shown in Table S2 in Multimedia Appendix 1. Two baseline variables, namely, employment status and pandemic-related disruption index, were not previously reported and can be seen in Table 1. Both variables appear to be balanced across the trial arms. Most parents of children were in paid employment and reported some level of COVID-19 pandemic–related disruption.

**Table 1:** Previously unreported baseline variables for randomized arms and overall.

Baseline variable			FAU^a^ arm (n=326)		Parent Positive arm (n=320)		Overall (N=646)
**Employment status^b^, n (%)**							
	In paid employment	251 (77.0)		256 (80.0)		507 (78.5)	
	Not in paid employment	75 (23.0)		64 (20.0)		139 (22.5)	
**Pandemic-related disruption index^c^, n (%)**							
	0	50 (15.3)		53 (16.6)		103 (15.9)	
	1	164 (50.3)		163 (50.9)		327 (50.6)	
	2 and 3^d^	112 (34.4)		104 (32.5)		216 (33.5)	

^a^FAU: follow-up as usual.

^b^Employment status was recoded into not in paid employment (at university, unable to work due to disability, homemaker or full-time parent, unemployed and seeking work, retired), and in paid employment (self-employed, part-time, full-time).

^c^Higher pandemic-related disruption index (a 3-level categorical variable) indicates more pandemic-related disruption.

^d^The 2 highest scores (2 and 3) in the pandemic-related disruption index were collapsed into 1 category.

### Moderation of Parent Positive Versus FAU Results on Clinical Outcomes

All results from univariate LMM are shown in Table S3 in Multimedia Appendix 1. Child gender, age, and ADHD symptoms, parental psychological distress, family conflict, household income, employment status, household overcrowding index, and pandemic-related disruption index did not significantly moderate the effects of Parent Positive on child conduct problems at T2 and T3 (Table S3 in Multimedia Appendix 1).

**Figure 1:**
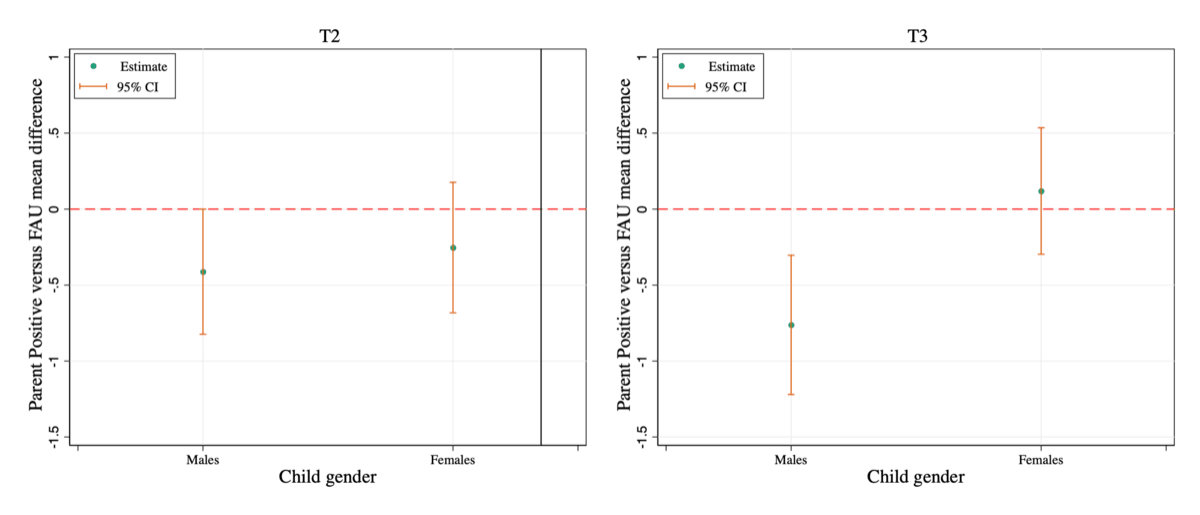
Moderation of intervention effects on Strengths and Difficulties Questionnaire child emotional problems by child gender at time points 2 (1-month) and 3 (2 -months). Lower scores on the y-axis indicate a positive difference (ie, lower severity of child emotional problems). FAU: follow-up as usual.

For child emotional problems, when accounting for other variables in the multivariate model, gender was a significant moderator of the Parent Positive versus FAU intervention effect at T2 and T3 (Table 2). Specifically, the Parent Positive versus FAU lower emotional problems effects were close to or significant in males only (T2: *B*=–0.41, 95% CI –0.82 to 0.0004; T3: *B*=–0.76, 95% CI –1.22 to –0.30), with this effect being significantly different in males as compared with females at T3, who did not show a significant reduction in emotional problems in the Parent Positive group (*B*=0.12, 95% CI –0.30 to 0.54; see Figure 1 and Table S4 in Multimedia Appendix 1). This result was similar to the moderation effect of child gender found in the univariate model (Figure S1 in Multimedia Appendix 1). No other significant moderation effects on child emotional problems at 1 and 2 months of follow-up were found. Candidate moderators that showed only significant main but not interaction effects (ie, no moderation) from the multivariate LMM are further discussed in Multimedia Appendix 1.

**Table 2:** Multivariate linear mixed-effects models for child conduct and emotional problems.

Baseline variable	SDQ^a^ Child conduct				SDQ Child emotion		
	*B*	2-Sided 95% CI	*P* value	*B*		2-Sided 95% CI	*P* value
Time^b^	0.01	–0.12 to 0.15	.82	0.52		–0.09 to 1.13	.10
Randomization arm^c^	–0.01	–0.24 to 0.22	.94	–0.57		–1.50 to 0.35	.23
Randomization arm ×^d^ time	–0.17	–0.39 to 0.05	.13	–1.07		–2.03 to –0.11	.03
SDQ Child conduct	0.64	0.57 to 0.72	<.001	N/A^e^		N/A	N/A
SDQ Child emotion	N/A	N/A	N/A	0.72		0.67 to 0.78	<.001
Child age	–0.01	–0.07 to 0.05	.84	–0.01		–0.09 to 0.08	.90
Child gender^f^	–0.23	–0.43 to –0.03	.03	0.01		–0.37 to 0.38	.98
Child gender × time	N/A	N/A	N/A	–0.45		–0.85 to –0.06	.02
Child gender × randomization arm	N/A	N/A	N/A	0.16		–0.43 to 0.75	.60
Child gender × time × randomization arm	N/A	N/A	N/A	0.72		0.12 to 1.33	.02
Household income^g^	0.07	–0.18 to 0.32	.59	–0.30		–0.66 to 0.06	.10
Overcrowding index^h^	0.01	–0.26 to 0.29	.93	–0.03		–0.56 to 0.50	.90
Employment status^i^	N/A	N/A	N/A	0.07		–0.25 to 0.38	.68
Family conflict	0.18	0.11 to 0.26	<.001	N/A		N/A	N/A
SDQ Child ADHD^j^ symptoms	0.09	0.05 to 0.13	<.001	0.09		0.04 to 0.14	<.001

^a^SDQ: Strengths and Difficulties Questionnaire.

^b^Reference category: time 2.

^c^Reference category: follow-up as usual arm.

^d^×: Interaction effect.

^e^N/A: not applicable (indicates absence of the covariate for the given model).

^f^Child gender (1: male, 2: female) was treated as continuous due to mean imputation (mean 1.49) conducted for 3 participants.

^g^Reference category: household income less than £29,999 (US $38,600.66) a year.

^h^Higher overcrowding index indicates more overcrowding at home.

^i^Reference category: not in paid employment.

^j^ADHD: attention-deficit/hyperactivity disorder.

## Discussion

### Principal Findings

Although research suggests that participant characteristics may impact the outcomes of parenting interventions, only a few studies have examined their moderating effects on child outcomes. Furthermore, the existing findings are often inconsistent and appear to depend on the specific intervention being studied [19,27]. As the Parent Positive app was developed to reverse children’s increases in conduct and emotional problems experienced during the COVID-19 pandemic, moderating effects within this context have not yet been examined. This study aimed to identify child, family, socioeconomic, and pandemic-related disruption factors at baseline that may significantly moderate Parent Positive effects on children’s conduct and emotional problems. The results showed that child gender was a significant moderator of the Parent Positive versus FAU effect on child emotional problems. None of the other investigated baseline factors played a moderating role in emotional problems, and no examined baseline factors were found to moderate effects on conduct problems.

This observed pattern of results in child conduct problems is similar to some previous research on face-to-face parenting interventions looking at child, family, and socioeconomic factors, indicating a lack of moderation effects [19,23,27]. However, it conflicts with some other studies suggesting significant moderation effects in the context of socioeconomic status [21], ADHD symptoms [41], and maternal depression [26]. Despite the growing emphasis on digital parenting interventions [12,13], the exploration of moderators in parenting interventions has predominantly centered on longer, face-to-face interventions guided by therapists [20]. This makes comparisons to most moderator studies challenging, considering the distinctive nature of Parent Positive as a short, smartphone app–based, and self-directed intervention, with an emphasis on fostering positive environments and experiences for both parents and children. Overall, these findings suggest that the Parent Positive effects on child conduct problems did not differ by the available participant characteristics. It should also be noted that a significant effect of Parent Positive versus FAU was not observed at 1- or 2-month follow-up on child conduct problems [15]. However, levels of conduct problems seemed to be decreasing at the 2-month follow-up after Parent Positive, possibly indicating that longer follow-ups may be necessary to see effects of the intervention on this particular outcome [42]. Subsequently, we might also see factors that moderate intervention effects.

After controlling for baseline emotional problems and other covariates, child gender was found to significantly moderate the effects of Parent Positive on child emotional problems at 1- and 2-month follow-up. While Parent Positive did not appear to be effective for females in terms of emotional problems, the intervention was effective for males. This reduction in emotional problem levels became more evident at the 2-month follow-up assessment. A similar effect was also found when examining moderation by child gender in the univariate model, enhancing reliability and robustness of this finding.

The finding of a moderation effect by child gender is similar to the finding of meta-analysis that looked at moderation of parenting interventions on child disruptive behaviors [20] but differs from findings in 2 RCTs [23,24]. This could be due to earlier studies predominantly involving males [43], whereas our sample had a more balanced representation of parents with male and female children. However, these prior studies examined the conduct problem outcome only, which might limit the comparability of the moderation effect on child emotional problems observed in our study. Notably, the emotional subscale of the SDQ used in this study encompasses dimensions such as anxiety and strongly aligns with anxiety measures across childhood [44], possibly allowing for meaningful comparisons with anxiety-focused treatments (eg, cognitive behavioral therapy). However, most studies in this domain have indicated that child gender does not moderate outcomes of treatments focusing on anxiety in children [45].

There are several other explanations. Parent Positive aimed to reduce child worry and enhance mood through positive parenting strategies while reducing harsh practices, aligning with evidence of parent training’s impact on child internalizing problems [46]. Notably, parents frequently accessed booster components of Parent Positive that focused on family-related processes, potentially improving the overall emotional atmosphere in families using Parent Positive [15]. The observed gender moderation may be explained by these boosters having a more significant impact on males. Existing research shows that parents are more likely to use positive parenting strategies for females [47,48] while harsh parenting practices for males [49]. Consequently, it is possible that male children, who may exhibit more externalizing emotions such as anger [50], might have responded more robustly than females to the positive changes in parenting introduced by the boosters. The potential for a greater shift in parenting practices toward males may have contributed to the enhanced effectiveness of Parent Positive for male children in addressing emotional problems.

However, the lack of a more consistent gender effect across previous studies prompts considering additional factors that might contribute to this variability. Variations in study methodologies, sample characteristics, and cultural contexts across different studies could account for the inconsistent findings [19]. In addition, while changes in parenting practices may influence emotional outcomes in male children, the relationship with conduct problems in males might be more complex and multifaceted. Exploring this variability in future research, along with the interplay between child gender, parenting changes, and conduct problems, could provide a more comprehensive understanding of the observed patterns.

Another possibility is that societal gender expectations of emotion expression may have influenced our findings. Brody’s theory [51] proposes that females are more likely to display internalizing emotions (eg, anxiety, sadness) while males tend to externalize emotions (eg, anger), which may contribute to the development of conduct problems [50]. These gender differences in emotional expression were supported by a meta-analytic review encompassing facial, behavioral, and vocal emotion expressions in children [52]. Given that our outcomes were parent-reported, and males are less likely to display internalizing emotions, it is important to consider the potential bias introduced by parents in accurately identifying and reporting emotions in males. Social expectations may also lead parents to anticipate and encourage less emotional expression in males than in females [51], possibly resulting in underreporting of emotional problems for male children [53]. To enhance the robustness of these findings, future research should consider incorporating additional measures of child emotional problems [53] from a range of informants (eg, teachers) and various technologies (eg, wearables that track heart rate variability as an indicator of anxiety).

### Strengths and Limitations

Participant and pandemic-related disruption factors were explored in this study as potential moderators of Parent Positive in a large general population sample within a cohort. Unlike previous research on moderators of parenting interventions, child outcomes of both conduct and emotional problems were examined, with the latter essential given the pandemic-related increase.

A few limitations should be noted. First, child outcomes were parent-reported. Despite their importance, bias may have occurred because parents were unblinded [54]. While blinding of participants was not possible due to the research design, results should be replicated using more objective approaches such as observational measures of child behavior conducted by a blinded researcher [55]. Second, the relatively short follow-up times, chosen for practical reasons, could potentially mask the emergence of moderation effects that may require a longer duration to manifest. In addition, the low sociodemographic variability may have decreased statistical power and diminished chances to detect moderation effects by sociodemographic characteristics. Furthermore, because of the lack of variability in some of the sociodemographic characteristics (eg, household income and employment status), moderation of effects was examined in a binary way, and it is important to acknowledge that reducing this range into 2 categories may pose a limitation, as the distinct experiences within each category might vary considerably. Overall, replications with longer follow-up times are necessary, including with more families from low socioeconomic backgrounds, and racially and ethnically minoritized families to improve generalizability.

Finally, SPARKLE used a Trial within a Cohort design and participants were recruited from the Co-SPACE cohort, which allowed for rapid recruitment but had some limitations, as it likely attracted families with interest and internet access [56]. The sample also had higher levels of child conduct and emotional problems and ADHD symptoms than both the UK national survey [55] and the wider Co-SPACE study sample [29]. While levels of parental psychological distress were also higher than expected in the general population [36], they did not differ from levels reported in the wider Co-SPACE sample [37]. In addition, not all relevant moderators of interest from prior research were included (eg, young parent age and initial parenting confidence), as we relied on the available variables within the Co-SPACE data set. Future research incorporating a wider array of moderators in future studies could provide a more comprehensive understanding of intervention outcomes.

### Conclusions

This study has demonstrated that Parent Positive has the potential to improve primary school-aged male children’s emotional problems in a wide range of families. The findings suggest that the effects of Parent Positive on emotional problems do not differ for children with different levels of ADHD symptoms, socioeconomic status and household crowding, family conflict, and parental psychological distress. Pandemic-related disruption was also not found to influence the effectiveness of the app on child emotional problems. There were no differential effects of Parent Positive on conduct problems by any of the participant characteristics and pandemic-related disruption circumstances, noting that overall, the app was not found to be effective in addressing child conduct problems in the main SPARKLE trial analysis [15]. While our findings suggest positive emotional outcomes for male children, the exploratory nature of this study, along with potential limitations in statistical power to detect moderated effects and limited variability in some potential moderating variables, warrants caution in drawing definitive conclusions. However, if the male sex advantage in effectiveness turns out to be replicated, the reasons for this need to be identified and steps taken to adapt Parent Positive to increase its value for females. This could involve explorations of differences in the way that parents think about and react to emotional problems in their male and female children and subsequent change to the app to take account of these differences. Additional future research should focus on multivariate moderation analyses and replicate these findings in larger and diverse samples, with longer follow-up times, and across different contexts and settings, to better understand the app’s effectiveness. In sum, while this study underscores the promising role of Parent Positive in fostering emotional well-being among male children, these findings are preliminary and continued research endeavors are essential to optimize its effectiveness and applicability in practice, especially in diverse familial contexts.

## Appendix

Multimedia Appendix 1 Additional details on statistical analysis, sample, clinical outcomes, and model results.

## Abbreviations

ADHD: attention-deficit/hyperactivity disorder
Co-SPACE: COVID-19: Supporting Parents, Adolescents, and Children During Epidemics
CONSORT: Consolidated Standards of Reporting Trials
DASS: Depression, Anxiety, and Stress Scale
FAU: follow-up as usual
LMM: linear mixed-effects models
RCT: randomized controlled trial
SDQ: Strengths and Difficulties Questionnaire
SPARKLE: Supporting Parents and Kids Through Lockdown Experiences
T1: baseline before randomization
T2: 1 month after randomization
T3: 2 months after randomization
