# Supplementary material for: Moderators of the Effects of a Digital Parenting Intervention on Child Conduct and Emotional Problems Implemented During the COVID-19 Pandemic: Results From a Secondary Analysis of Data From the Supporting Parents and Kids Through Lockdown Experiences (SPARKLE) Randomized Controlled Trial
Source: JMIR Pediatr Parent. 2024 Oct 8;7:e53864. doi: 10.2196/53864 (PMC11496916; doi:10.2196/53864)
Supplement: Multimedia Appendix 1 [file pediatrics_v7i1e53864_app1.docx]

## Original Paper

# Moderators of the Effects of a Digital Parenting Intervention on Child Conduct and Emotional Problems Implemented During the COVID-19 Pandemic: Results From a Secondary Analysis of Data From the SPARKLE Randomized Controlled Trial

# Multimedia Appendix 1

### Online Supplement

### A list of steps in the statistical analysis of child emotional problems

1. Base Model (LMM):

- The base model can be represented by the following equation.

Y = β0 + β1 Intervention arm + β2 Time + β3 (Intervention arm × Time) + β4 Baseline child emotional problems + β5 Child age + β6 Child gender + β7 Household income + β8 Overcrowding index + β9 Employment status + ϵ

1. Univariate Linear Mixed-Effects Models:

- Fit LMM for each baseline moderator variable of interest as an independent variable in the base model background. Note that we don’t know if a variable is a moderator unless the interaction term in the second equation is significant, but we use that term for consistency. The regression equation example is:

Y = β0 + β1 Intervention arm + β2 Time + β3 (Intervention arm × Time) + β4 Baseline child emotional problems + β5 Child age + β6 Child gender + β7 Household income + β8 Overcrowding index + β9 Employment status + β10 Moderator + ϵ

- Fit LMM models for each baseline variable of interest as an independent moderator to the model background. The regression equation example is:

Y = β0 + β1 Intervention arm + β2 Time + β3 (Intervention arm × Time) + β4 Baseline child emotional problems + β5 Child age + β6 Child gender + β7 Household income + β8 Overcrowding index + β9 Employment status + β10 Moderator + β11 (Intervention arm × Time × Moderator) + ϵ

1. Multivariate Linear Mixed-Effects Models:

- Fit forward stepwise LMM with identified main effects from univariate models.
- Add main effects and 3-way interaction terms in a stepwise manner. The regression equation example is:

Y = β0 + β1 Intervention arm + β2 Time + β3 (Intervention arm × Time) + β4 Baseline child emotional problems + β5 Child age + β6 Child gender + β7 Household income + β8 Overcrowding index + β9 Employment status + β10 Moderator 𝑥*a* + β11 (Intervention arm × Time × Moderator 𝑥*a*) + β12 Moderator 𝑥*b* + β13 (Intervention arm × Time × Moderator 𝑥*b*) + … + ϵ

Table S1. Sample characteristics^a^ by randomized arms and overall at baseline.

| Characteristic | |  | FAU^b^ Arm | *Parent Positive* Arm | Overall |
| --- | --- | --- | --- | --- | --- |
|  |  |  | (n=326) | (n=320) | (N=646) |
| **Child age (years)***,* mean (SD) | |  | 7.52 (1.65) | 7.38 (1.68) | 7.45 (1.67) |
| **Child gender,** n (%) | |  |  |  |  |
|  | Male |  | 172 (52.8) | 158 (49.4) | 330 (51.1) |
|  | Female |  | 152 (46.6) | 161 (50.3) | 313 (48.5) |
|  | Prefer not to say |  | 2 (0.6) | 1 (0.3) | 3 (0.5) |
| **Household income** ^c^, n (%) | |  |  |  |  |
|  | Less than £29,999 ($36,769.5) a year |  | 73 (22.4) | 60 (18.8) | 133 (20.6) |
|  | Greater than £30,000 ($36,770.7) a year |  | 236 (72.4) | 241 (75.3) | 477 (73.8) |
|  | Prefer not to say |  | 16 (4.9) | 16 (5.0) | 32 (5.0) |
|  | Missing |  | 1 (0.3) | 3 (0.9) | 4 (0.6) |
| **Household overcrowding**, n (%) | |  |  |  |  |
|  | Lower |  | 289 (88.7) | 278 (86.9) | 567 (87.8) |
|  | Higher |  | 36 (11.0) | 40 (12.5) | 76 (11.8) |
|  | Missing |  | 1 (0.3) | 2 (0.6) | 3 (0.5) |
| **Isolation status,** n (%) | |  |  |  |  |
|  | Living life as normal |  | 78 (23.9) | 80 (25) | 158 (24.5) |
|  | Social distancing |  | 241 (73.9) | 230 (71.9) | 471 (72.9) |
|  | Self-isolating |  | 7 (2.1) | 10 (3.1) | 17 (2.6) |
| **Local lockdown (last month),** n (%) | |  |  |  |  |
|  | No |  | 207 (63.5) | 210 (65.6) | 417 (64.6) |
|  | Yes |  | 119 (36.5) | 110 (34.4) | 229 (35.4) |
| **Home working status,** n (%) | |  |  |  |  |
|  | At home |  | 89 (27.3) | 114 (35.6) | 203 (31.4) |
|  | Out of the home |  | 63 (19.3) | 44 (13.8) | 107 (16.6) |
|  | Both |  | 54 (16.6) | 49 (15.3) | 103 (15.9) |
|  | Not working |  | 111 (34.0) | 96 (30.0) | 207 (32.0) |
|  | Missing |  | 9 (2.8) | 17 (5.3) | 26 (4.0) |
| **Physical school attendance (last week),** n (%) | | |  |  |  |
|  | No |  | 28 (8.6) | 24 (7.5) | 52 (8.0) |
|  | Yes |  | 297 (91.1) | 295 (92.2) | 592 (92.1) |
|  | Missing |  | 1 (0.3) | 1 (0.3) | 2 (0.3) |
| **Access to outside space,** n (%) | |  |  |  |  |
|  | No |  | 19 (5.8) | 12 (3.8) | 31 (4.8) |
|  | Yes |  | 307 (94.2) | 308 (96.3) | 615 (95.2) |
| **Family Conflict,** n (%) | |  |  |  |  |
|  | mean (SD) |  | 3.11 (1.75) | 3.1 (1.72) | 3.11 (1.73) |
|  | Missing |  | 12 (3.68) | 11 (3.44) | 23 (3.56) |
| **DASS-21**^d^**,** Median (IQR) | |  | 29 (16-48) | 24 (12-43) | 26 (12-46) |
| **SDQ**^e^ **Child ADHD**^f^ **symptoms**, mean (SD) | | | 5.37 (2.90) | 5.23 (2.70) | 5.30 (2.80) |

^a^If data was incomplete, a row describing missingness was added.

^b^FAU: follow-up as usual.

^c^Household income was recoded into less than £29,999 ($36,769.5) a year and greater than £30,000 ($36,770.7) a year.

^d^DASS-21: Depression, Anxiety, Stress Scale–21 Items.

^e^SDQ: Strengths and Difficulties Questionnaire.

^f^ADHD: attention-deficit/hyperactivity disorder.

Table S2. Summary of clinical outcomes^a^ at baseline, time point 2 and 3.

| Outcome variable | Baseline | | | Time point 2 | | | Time point 3 | | |
| --- | --- | --- | --- | --- | --- | --- | --- | --- | --- |
|  | FAU^b^ arm  (n=326) | *Parent Positive* arm  (n=320) | Overall  (N=646) | FAU arm  (n=326) | *Parent Positive* arm  (n=320) | Overall  (N=646) | FAU arm  (n=326) | *Parent Positive* arm (n=320) | Overall  (N=646) |
| **SDQ**^c^ **Child conduct problems** |  |  |  |  |  |  |  |  |  |
| Mean (SD)  Missing, n (%) | 2.71 (2.05) | 2.58 (1.93) | 2.64 (1.99) | 2.55 (2.01) | 2.48 (2.04) | 2.55 (2.02) | 2.50 (2.10) | 2.19 (1.77) | 2.37 (1.97) |
|  |  |  |  | 60 (18.40) | 120 (37.50) | 180 (27.90) | 70 (21.50) | 134 (41.90) | 204 (31.60) |
| **SDQ Child emotional problems** |  |  |  |  |  |  |  |  |  |
| Mean (SD)  Missing, n (%) | 3.88 (2.66) | 3.66 (2.63) | 3.77 (2.65) | 3.71 (2.66) | 3.21 (2.59) | 3.49 (2.64) | 3.45 (2.62) | 3.03 (2.52) | 3.27 (2.58) |
|  |  |  |  | 60 (18.40) | 120 (37.50) | 180 (27.90) | 70 (21.50) | 134 (41.90) | 204 (31.60) |

^a^If data was incomplete, a row describing missingness was added.

^b^FAU: follow-up as usual.

^c^SDQ: Strengths and Difficulties Questionnaire.

### Child Conduct Problems

#### Identifying Predictors and Moderators of Intervention Effects in Univariate Mixed-Effects Models

Results from all of the univariate LMM for child conduct problems are presented in Table S3. After controlling for covariates in univariate LMM, child gender, ADHD symptoms, parental psychological distress, and family conflict were significant predictors of child conduct problems across the two follow-up time points. However, this finding was irrespective of the intervention arm, that is, no moderation was found.

Table S3. Univariate linear mixed-effects models^a^ for child conduct and emotional problems.

| Baseline variable | SDQ^b^ Child conduct | | | SDQ Child emotion | | |
| --- | --- | --- | --- | --- | --- | --- |
|  | B | 2-sided 95% CI | *P* value | B | 2-sided 95% CI | *P* value |
| **Without the interaction term** |  |  |  |  |  |  |
| Child age | –0.01 | –0.07 to 0.06 | .84 | –0.01 | –0.09 to 0.06 | .72 |
| Child gender^c^ | –0.32 | –0.52 to –0.11 | .003 | –0.10 | –0.36 to 0.16 | .47 |
| SDQ Child ADHD^d^ symptoms | 0.10 | 0.06 to 0.14 | <.001 | 0.09 | 0.04 to 0.14 | <.001 |
| Parental psychological distress | 0.01 | 0.00 to 0.01 | .003 | 0.00 | 0.00 to 0.01 | .14 |
| Family conflict | 0.19 | 0.12 to 0.27 | <.001 | 0.00 | –0.08 to 0.09 | .96 |
| Household income^e^ | 0.03 | –0.25 to 0.30 | .86 | –0.32 | –0.68 to 0.04 | .08 |
| Overcrowding index^f^ | 0.08 | –0.24 to 0.39 | .63 | 0.02 | –0.37 to 0.41 | .93 |
| Employment status^g^ | 0.18 | –0.08 to 0.44 | .18 | 0.08 | –0.25 to 0.41 | .64 |
| COVID-related disruption index^h^ | N/A^i^ | N/A | .46 | N/A | N/A | .61 |
|  |  |  |  |  |  |  |
| **With the interaction term** |  |  |  |  |  |  |
| Child age | 0.02 | –0.08 to 0.11 | .73 | –0.06 | –0.18 to 0.06 | .33 |
| Child age X^j^ time X randomization arm | 0.09 | –0.04 to 0.22 | .17 | –0.15 | –0.33 to 0.03 | .11 |
| Child gender | –0.28 | –0.59 to 0.03 | .08 | –0.11 | –0.51 to 0.29 | .58 |
| Child gender X time X randomization arm | 0.10 | –0.33 to 0.53 | .66 | 0.73 | 0.12 to 0.34 | .02 |
| SDQ Child ADHD symptoms | 0.11 | 0.05 to 0.17 | <.001 | 0.09 | 0.02 to 0.16 | .01 |
| SDQ Child ADHD symptoms X time X randomization arm | –0.02 | –0.10 to 0.06 | .63 | –0.04 | –0.15 to 0.07 | .45 |
| Parental psychological distress | 0.01 | 0.00 to 0.02 | .01 | 0.01 | 0.00 to 0.02 | .03 |
| Parental psychological distress X time X randomization arm | 0.00 | –0.01 to 0.00 | .32 | 0.00 | –0.01 to 0.02 | .70 |
| Family conflict | 0.18 | 0.08 to 0.28 | <.001 | 0.00 | –0.12 to 0.12 | .99 |
| Family conflict X time X randomization arm | –0.09 | –0.23 to 0.04 | .16 | –0.11 | –0.30 to 0.08 | .24 |
| Household income | 0.07 | –0.32 to 0.46 | .72 | –0.53 | –1.03 to –0.02 | .04 |
| Household income X time X randomization arm | 0.19 | –0.38 to 0.77 | .51 | –0.67 | –1.49 to 0.15 | .11 |
| Overcrowding index | 0.14 | –0.35 to 0.63 | .58 | –0.68 | –1.31 to –0.04 | .04 |
| Overcrowding index X time X randomization arm | 0.23 | –0.39 to 0.85 | .47 | –0.84 | –1.72 to 0.04 | .06 |
| Employment status | 0.25 | –0.12 to 0.62 | .18 | –0.18 | –0.65 to 0.30 | .46 |
| Employment status X time X randomization arm | –0.13 | –0.69 to 0.44 | .66 | –0.14 | –0.94 to 0.67 | .74 |
| Pandemic-related disruption index | N/A | N/A | .26 | N/A | N/A | .35 |
| Pandemic-related disruption index X time X randomization arm^k^ | N/A | N/A | .77 | N/A | N/A | .09 |

^a^All models included the following covariates: baseline outcome, randomization arm, time, time-randomization arm interaction, child age, child gender, household income, and overcrowding index; and employment status in child emotional outcome models.

^b^SDQ: Strengths and Difficulties Questionnaire.

^c^Child gender (1: male, 2: female) was treated as continuous due to mean imputation (mean 1.49) conducted for 3 participants.

^d^ADHD: attention-deficit/hyperactivity disorder.

^e^Reference category: household income less than £29,999 ($36,769.5) a year.

^f^Higher overcrowding index indicates more overcrowding at home.

^g^Reference category: not in paid employment.

^h^Higher pandemic-related disruption index indicates more pandemic-related disruption.

^i^N/A: not applicable.

^j^X: Interaction effect.

^k^Omnibus *P v*alue is reported for pandemic-related disruption index (3-level categorical variable).

#### Identifying Predictors of Intervention Effects in Multivariate Mixed-Effects Models

Several variables were predictors, but not moderators, of child conduct problem outcomes. Results are displayed in Table 2 in the main body of the manuscript.

After controlling for covariates in the multivariate model, child ADHD symptoms and family conflict were significant predictors of child conduct problems. Specifically, children with more ADHD symptoms and greater family conflict at baseline exhibited significantly higher levels of conduct problems across T2 and T3. Child gender, a variable included in the base model, was also a predictor, with females displaying lower levels of conduct problems compared to males across T2 and T3.

### Child Emotional Problems

#### Identifying Predictors and Moderators of Intervention Effects in Univariate Mixed-Effects Models

Table S3 presented above displays the univariate LMM results. After controlling for covariates in univariate LMM, child ADHD symptoms and the overcrowding index were found to be significant predictors of child emotional problems across the two follow-up time points.

A moderation effect marked by a significant interaction between child gender, intervention arm, and time was also observed at T2 and T3 in the univariate model. Specifically, the forest plot (Figure S1) shows that the *Parent Positive* versus FAU effects in the univariate model were only significant in males, with a significantly larger effect in males as compared to females at T3.

Figure S1. Moderation of intervention effects on SDQ Child emotional problems by child gender at time point 2 (1 month) and 3 (2 months) in a univariate mixed-effects model.

FAU: follow-up as usual. SDQ: Strengths and Difficulties Questionnaire.

#### Identifying Predictors in Multivariate Mixed-Effects Models

Several variables showed significant main effects, i.e., were predictors of outcome regardless of intervention allocation, on child emotional problem outcomes. Results are displayed in Table 2 in the main body of the manuscript.

After controlling for covariates in the multivariate model, child ADHD symptoms were identified as a significant predictor. Irrespective of the intervention arm, children with more ADHD symptoms at baseline had significantly higher levels of emotional problems across T2 and T3. No other significant main effects/predictors were found in the multivariate model. Moderation results from the multivariate model are displayed in the main body of the manuscript.

Table S4. Moderation of the effects of *Parent Positive* vs FAU in univariate and multivariate linear mixed-effects models for SDQ^a^ Child emotional problems at time point 2 and 3.

| Baseline moderator variable | | Time point 2 | | | Time point 3 | | |
| --- | --- | --- | --- | --- | --- | --- | --- |
|  |  | B | 2-sided 95% CI | *P* value | B | 2-sided 95% CI | *P* value |
| **Univariate models** | |  |  |  |  |  |  |
| Child gender X^b^ time X randomization arm | |  |  |  |  |  |  |
|  | Males | –0.42 | –0.83 to 0.005 | .047 | –0.77 | –1.19 to –0.35 | <.001 |
|  | Females | –0.25 | –0.69 to 0.18 | .25 | 0.12 | –0.32 to 0.57 | .58 |
| Overcrowding index^c^ X time X randomization arm | |  |  |  |  |  |  |
|  | 25th percentile | –0.55 | –0.91 to –0.20 | .002 | –0.38 | –0.74 to –0.01 | .04 |
|  | 50th percentile | –0.40 | –0.70 to –0.09 | .01 | –0.36 | –0.67 to –0.05 | .02 |
|  | 75th percentile | –0.27 | –0.57 to 0.04 | .09 | –0.34 | –0.66 to –0.03 | .03 |
| **Multivariate models** | |  |  |  |  |  |  |
| Child gender X time X randomization arm | |  |  |  |  |  |  |
|  | Males | –0.41 | –0.82 to 0.0004 | .05 | –0.76 | –1.22 to –0.30 | <.001 |
|  | Females | –0.25 | –0.68 to 0.18 | .25 | 0.12 | –0.30 to 0.54 | .57 |

^a^SDQ: Strengths and Difficulties Questionnaire.

^b^X: Interaction effect.

^c^Higher overcrowding index indicates more overcrowding at home
